# Pose-invariant matching for non-rigid 3D models using Isomap

**DOI:** 10.1371/journal.pone.0264192

**Published:** 2022-03-16

**Authors:** Hairong Jin, Haichao Huang, Zhiqiang Wang, Yuqing Xie, Xinyue Zhou, Liming Huang, Zhouzhenyan Hong

**Affiliations:** 1 Polytechnic Institute, Zhejiang University, Ningbo, Zhejiang, China; 2 State Grid Zhejiang Electric Power Corporation Information & Telecommunication Branch, Hangzhou, Zhejiang, China; 3 State Grid Hangzhou Power Supply Company, Hangzhou, Zhejiang, China; 4 Zhejiang Huayun information technology co., ltd, Hangzhou, Zhejiang, China; University of Glasgow, UNITED KINGDOM

## Abstract

The wide usage of 3D mesh models greatly increases the importance of an effective matching algorithm for them. In this paper, we propose a novel 3D model matching algorithm. Firstly, vertices on the input 3D mesh models are mapped to 1D space by employing Isomap. A pose-invariant feature set is then constructed from the vertices in 1D space. Finally, the similarity between any two 3D models can be computed by comparing their feature sets. Experimental results show that the algorithm is not only invariant to translation, rotation, scaling, but also invariant to different poses of 3D models. Additionally, the algorithm is robust to noise.

## Introduction

With the explosion of 3D models and the expansion of 3D model databases, 3D model database management and 3D model matching and retrieval [1, 2] become more and more significant. An efficient algorithm is significant for solving problems in various areas [3–5]. Efficient and accurate retrieval of 3D models has become a research hotspot for many years. 3D model retrieval, given a retrieval object, to find the matching 3D models in the 3D model database. Some 3D model retrieval algorithms use images to query 3D models from the database [6]. Others use 3D models as the retrieval object to match 3D models from the database by calculating the similarity between the two models [1]. Tangelder et al. [7] survey several content-based 3D model retrieval algorithms based on surface models and volume models and introduce the requirement of evaluation of content-based 3D model retrieval algorithms. For the feature-based method, it is crucial whether the descriptors have the invariance of the poses, rotation, and scale of the 3D model, directly affecting the accuracy of 3D model retrieval.

Recent advances in 3D model retrieval, deep learning algorithms are used to match 3D models in databases [8]. Deep learning algorithms rely on tremendous training data to obtain good performance. However, acquiring high-quality 3D models is difficult, which demands high costs. The absence of 3D model training data will lead to the decline in the accuracy of 3D model matching.

**Fig 1:**
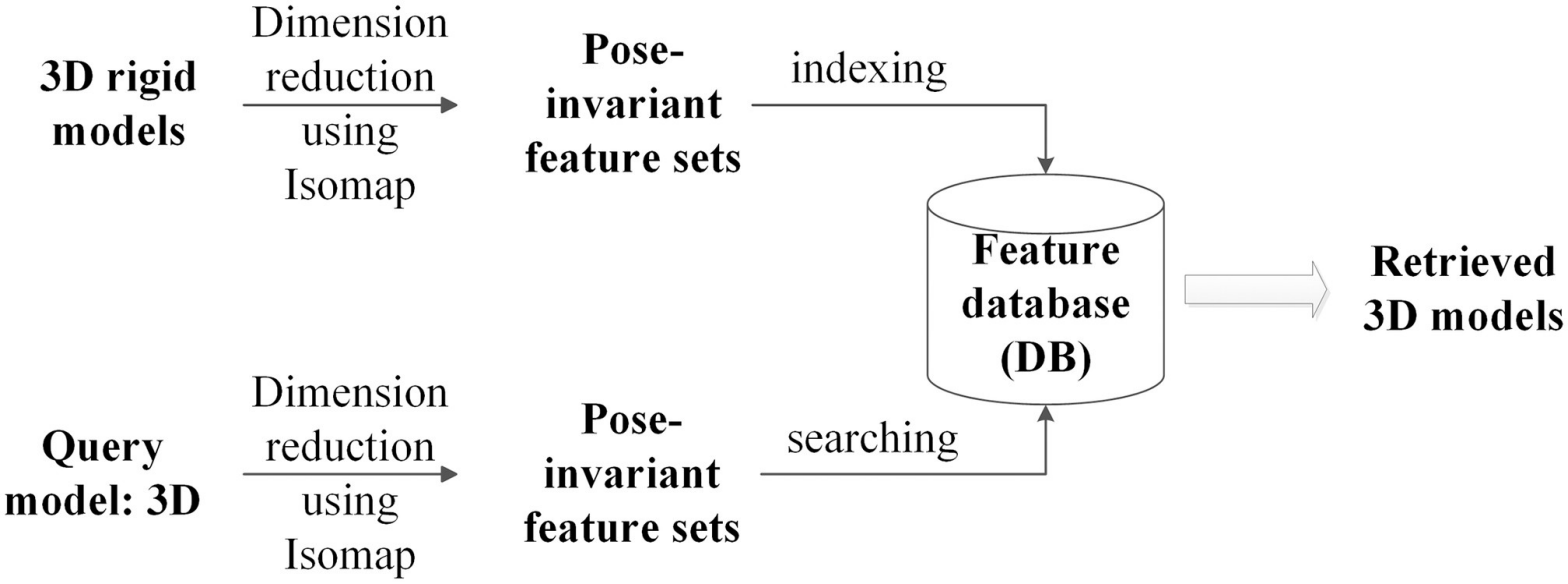
Overview of the 3D model retrieval process using our method.

In this paper, we propose a novel 3D model matching algorithm for non-rigid models. As described in Fig 1, a new 3D model retrieval approach can be constructed based on the algorithm. The algorithm maps all vertices on 3D mesh models to 1-dimensional linear space with Isomap first. Secondly, we construct pose-invariant feature sets with coordinate vectors of vertices in 1-dimensional space. At last, we compute the similarity measure between two 3D models by comparing the two pose-invariant feature sets. Benefit from the little computation required for executing Isomap, the algorithm behaves very efficiently.

Our method is evaluated on a 35 3D model dataset. The 35 3D models are from the SHREC 2011 non-rigid 3D shape dataset [9]. The experimental results show that the algorithm is not only invariant to translation, rotation, and scaling, but also invariant to different poses. Additionally, it is robust against geometrical noise. We also compare the performance of our method and the other six methods through precision vs. recall curves. The comparison experimental results show that our method can achieve better performance than other methods, including D2 [10], Adaptive Shape Feature [11], LightField Descriptor [12], Spherical Harmonic Representation [13]. Furthermore, our method performs better than Pointnet [14] and Pointnet++ [15] with the absence of training data.

Our contributions are two-fold:

- Our method measures similarity between two 3D models with pose-invariant feature sets. Then we can obtain high performance in 3D model retrieval without training data through this method.
- Pose-invariant feature sets invariant to translation, rotation, scaling, poses, and geometrical noise of 3D models. Thus, our method is applicable for non-rigid 3D models especially.

The remainder of the paper is organized as follows. At first, we discuss the related work of 3D model retrieval. Then, we introduce Isomap briefly. In the algorithm is described in detail. After that, we show some experimental results of the algorithm and comparisons with other 3D model retrieval algorithms. Finally, we conclude this paper and point out further work.

## Related work

In this section, we introduce various algorithms and technologies related to 3D model retrieval. At first, we introduce some methods for 3D model matching. Secondly, we review some deep learning algorithms for 3D model retrieval. Eventually, we discuss the shortcomings of existing methods.

With the wide usage of 3D models in the industry, searching similar models in 3D model databases accurately and quickly becomes a more and more important problem. Recently, it has attracted many researchers to discover an efficient way for content-based 3D model retrieval. Yang [16] defines that a basic content-based 3D model retrieval system should contain several parts, including feature extraction, similarity search, and the query interface. In the retrieval phase, the matching of two 3D models is achieved by comparing the feature descriptors. Therefore, finding appropriate feature descriptors and metrics to measure the similarities between any two different 3D models is the key to the 3D model retrieval. The feature descriptor of a 3D model can be represented by using a shape distribution [10], spherical harmonics [13], light field [12], or spectral approaches [17]. Bespalov et al. use hierarchy scale-space representations [18] as feature descriptors to compare models. Ip and Regli [19] combine a curvature-based shape descriptor with support vector machines (SVM) to compare different models. Hou et al. [20] use shape information to cluster the semantics of parts by SVMs to learn 3D shapes for a system to recognize 3D models. A recent survey about relevant methods is given in Reference [21].

For the past few years, feature descriptors are also found with lower-dimensional attributes by using dimension reduction approaches, such as Principal Component Analysis (PCA) [22]. Although PCA keeps most of the information on 3D models, it fails to maintain their non-linear structures. Isometric feature mapping (Isomap) [23] is a dimension reduction approach that can successfully discover the non-linear structure.

In recent years, deep learning [24] has been widely applied to various fields and achieved outstanding results. Many researchers have applied deep learning to 3D model retrieval and proposed many 3D model retrieval algorithms based on deep learning. Qi [14, 15] proposed PointNet and its improved version, PointNet++, which can be applied to 3D points cloud models classification and segmentation. This method can learn the features of the points cloud data directly and then realize the classification and segmentation of the 3D points cloud models. 3D model retrieval based on multiple view images [25] has been widely researched recently. Su [26] trained the convolutional neural network with images of different perspectives of 3D models and proposed the multi-view convolutional neural network (MVCNN). This method integrates multi-view features to realize 3D model recognition. Asko [27] proposed RotationNet based on the convolutional neural network. In this method, the multi-view images of 3D models are used as the inputs of the convolutional neural network. The labels of the viewpoints are used as the latent variables to learn in an unsupervised way on the basis of MVCNN to realize the classification and pose estimation of 3D models. Compared with MVCNN, this method only needs a part of multi-view images for inference. Nie [28] proposed a novel multi-branch graph convolution network (M-GCN) to realize the 3D model retrieval based on 2D images. Liu [29] proposed a cross-domain 3D model retrieval method based on visual domain adaptation. Su [30] decomposed the multi-view graph-based similarity measures into multiple single-view graph-based similarity measures and proposed a hierarchical graph structure learning method (HGS) to address 3D model retrieval.

However, the 3D model retrieval algorithms based on deep learning learn features of data directly, which has certain randomness and unreliability. Moreover, supervised learning algorithms with high accuracy in deep learning often need a large amount of data as training samples. Furthermore, Most traditional approaches are focused on measuring rigid models and failed to identify non-rigid models well. For non-rigid models, an ideal shape matching algorithm should be not only invariant to basic geometry transformations, including translation, rotation, and scaling, but also invariant to different poses. That means two models which are different in the pose but represent the same object should be recognized as similar models. Additionally, it would be better if the algorithm is robust enough against noise data.

## Isomap

Dimension reduction methods can be applied to 3D models to find the feature from lower dimension attributes while keeping most of the information of 3D models. There are many methods to reduce the dimensionality of 3D models, such as Principal component analysis (PCA), Multidimensional scaling (MDS), Isomap, etc. Both PCA and MDS are easier to implement, efficiently computable, and guaranteed to find the linear structure of the input data points. In fact, they all try to find a low-dimensional embedding of the data points. However, there are also significant differences between PCA and MDS. The most important one is that PCA tries to keep the variance of the data points, while MDS tries to preserve the Euclidean distance of the data points. The common drawback of PCA and MDS is that they cannot find the non-linear structure of the data points. For data points on a manifold, both methods fail to detect the true degrees of freedom of them. Isomap [23], as a non-linear dimensionality reduction method, improves the traditional MDS and is proved to be more suitable to deal with data points sampled from a manifold. Given a data point set, the main goal of Isomap is to reduce the dimensionality of the input data points. Meanwhile, it tries to make the Euclidean distances between vertices in low-dimensional space equal to the geodesic distances in high-dimensional space. Isomap not only keeps the salient parts of 3D models, but also preserves the geometrical structure of them. The main steps of Isomap include measuring the geodesic distances between all pairs of points on the manifold *M*, which those data points resided on, and then using MDS to find a low-dimensional embedding that keeps the geodesic distances instead of Euclidean distances. Let *D*_*M*_ be the matrix of geodesic distances {*d*_*M*_(*i*, *j*)}, where *d*_*M*_(*i*, *j*) is the geodesic distance between any pair of data points *v*_*i*_ and *v*_*j*_ on *M*, the goal of Isomap is to find data points *y*_*i*_ and *y*_*j*_ in low dimensional space *Y*, such that the points in *Y* globally minimize a cost function

$$\begin{array}{c} E=\|\tau \left(D_{M}\right)-\tau \left(D_{Y}\right)\|, \end{array}$$ (1)

where *D*_*Y*_ denotes the matrix of Euclidean distances {*d*_*Y*_(*i*, *j*) = ‖ *y*_*i*_ − *y*_*j*_ ‖} and *τ* is an operator converting distances to inner products that can be used to uniquely characterize the geometry of the data. The minimum of the Eq (1) can be achieved by setting the coordinates *y*_*i*_ to the top *d* eigenvectors of the matrix *τ*(*D*_*M*_), where *d* is the dimensionality of the space *Y*. More details can be found in Reference [23].

## Algorithm

### Preprocessing input models to the same vertices count

**Fig 2:**
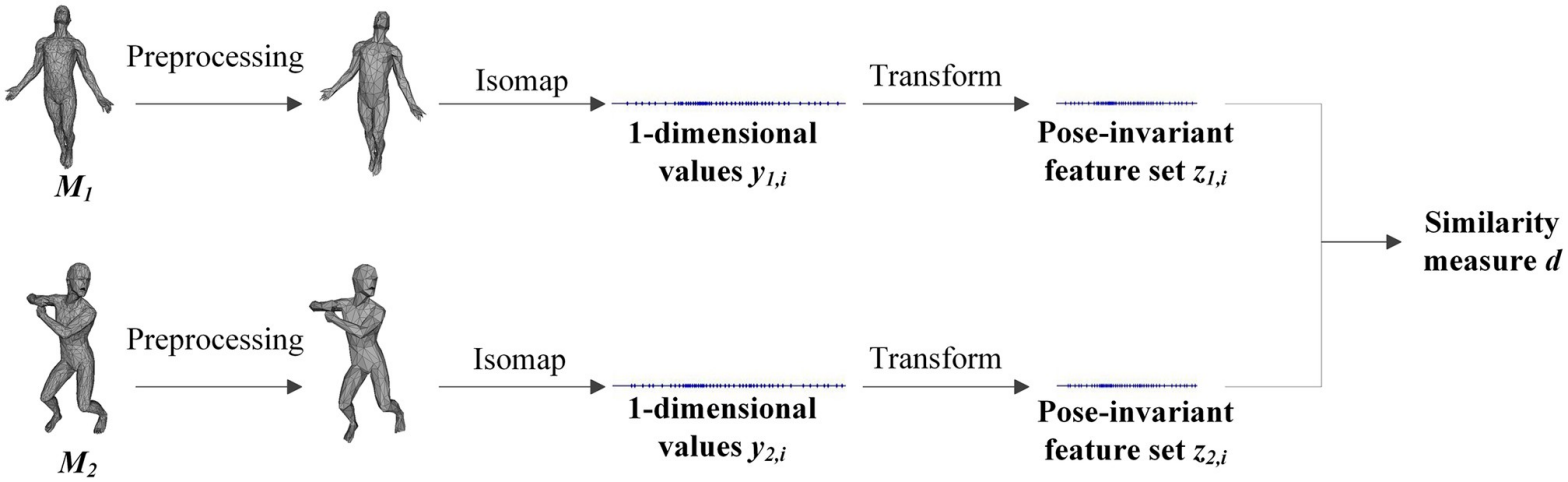
An overview of our algorithm.

In this section, we will briefly describe our 3D model matching algorithm. Let *M*_1_ and *M*_2_ be any two 3D models. The goal of our algorithm is to fast compute the similarity between them. Fig 2 shows an overview of the algorithm. It consists of 5 steps. The first step is to preprocess 3D models *M*_1_ and *M*_2_ to contain the same vertices count. The second one is to find the matrices of geodesic distances $D_{M_{1}}=\left\{d_{M_{1}}\left(i,j\right)\right\}$ and $D_{M_{2}}=\left\{d_{M_{2}}\left(i,j\right)\right\}$ for *M*_1_ and *M*_2_, where $d_{M_{1}}\left(i,j\right)$ and $d_{M_{2}}\left(i,j\right)$ denote the geodesic distances between any two different vertices *V*_*i*_ and *V*_*j*_ on the models respectively. In the third step, classical MDS is applied to *M*_1_ and *M*_2_ respectively to find embeddings of the vertices in a 1-dimensional Euclidean space *Y* that best preserves the geodesic distances $D_{M_{1}}$ and $D_{M_{2}}$. That is, each vertex *V*_*i*_ is mapped to a scalar value *y*_*i*_. And then, two feature sets {*z*_1,*i*_} and {*z*_2,*i*_} are obtained for *M*_1_ and *M*_2_ respectively by processing these scalar values in the fourth step. The final step is to measure the similarity between *M*_1_ and *M*_2_ by comparing feature sets {*z*_1,*i*_} and {*z*_2,*i*_}.

The first step of our algorithm is to preprocess 3D models *M*_1_ and *M*_2_ to make them contain the same count vertices. Assuming that *M*_1_ and *M*_2_ contain *m*_1_ and *m*_2_ vertices count respectively, then we simplify the models *M*_1_ and *M*_2_, such that they contain *m* vertices, where *m* is number given by the user and *m* ≤ *min*{*m*_1_, *m*_2_} (In this paper, m is set to be 500 because the vertices counts of the models used to test our algorithm are all more than 500). There are many mesh model simplification algorithms, such as QEM [31], etc. Due to its’ high fidelity maintenance and high performance compared with other mesh model simplification algorithms, we adopt the QEM algorithm to simplify input models here.

### Geodesic distances matrix construction

Computing the exact geodesic distance between two different vertices *V*_*i*_ and *V*_*j*_ is a time-consuming task in computer graphics. The classical algorithm [32] proposed by Chen and Han has *O*(*n*^2^ *log*(*n*)) worst-case time complexity. Recently, Xing and Wang [33] proposed a new algorithm that improved it in performance, and the computing time is greatly reduced in many examples. Our algorithm employs Xing and Wang’s algorithm to construct geodesic distances matrix $D_{M_{1}}=\left\{d_{M_{1}}\left(i,j\right)\right\}$ and $D_{M_{2}}=\left\{d_{M_{2}}\left(i,j\right)\right\}$, where $d_{M_{1}}\left(i,j\right)$ and $d_{M_{2}}\left(i,j\right)$ denotes the geodesic distance between any two different vertices *V*_*i*_ and *V*_*j*_ on models respectively.

### Finding an embedding of all vertices in 1-dimensional Euclidean space

For Isomap can be regarded as a geodesic version of MDS (Multidimensional Scaling), the next step of Isomap is applying MDS to the input data after obtaining geodesic distances. MDS is one of the commonly used methods to reduce the given data points’ dimensionality. The goal is to find a low-dimensional embedding that preserves the interpoint distances. Given a vertex set {*V*_*i*_} in 3D space and a distance matrix *D*_*M*_ = {*d*_*M*_(*i*, *j*)}, MDS tries to find a mapping *R*, which maps every 3D vertices *V*_*i*_ to the corresponding 1D scalar values *y*_*i*_, such that the cost function *E* = ∑_*i,j*_(*d*_*M*_(*i,j*) − *τ*(*i,j*))^2^, where *τ*(*i*, *j*) denotes the Euclidean distance between *y*_*i*_ and *y*_*j*_ in 1D space. Our algorithm uses MDS to map all vertices on models *M*_1_ and *M*_2_ to 1D Euclidean space *Y* respectively.

### Obtaining pose-invariant feature sets

Let {*y*_1,*i*_} and {*y*_2,*i*_} denote the 1D scalar values obtained by applying Isomap to model *M*_1_ and *M*_2_ respectively in the previous step, the following transforms are then performed on them to obtain the feature sets which are used to measure the similarity between models.

1. The set {*y*_1,*i*_} and {*y*_2,*i*_} are sorted in ascending order such that *y*_1,1_ ≤ *y*_1,2_ ≤ … ≤ *y*_1,*n*_ and *y*_2,1_ ≤ *y*_2,2_ ≤ … ≤ *y*_2,*n*_.
2. Let $y_{1,i}^{{}^{\prime }}=y_{1,i}-\frac{1}{n}\sum_{j=1}^{n}y_{1,j}$ and $y_{2,i}^{{}^{\prime }}=y_{2,i}-\frac{1}{n}\sum_{j=1}^{n}y_{2,j}$, *i* = 1, …, *n*. The purpose of this transform is to make $\left\{y_{1,i}^{{}^{\prime }}\right\}$ and $\left\{y_{2,i}^{{}^{\prime }}\right\}$ translation-invariant to input models.
3. Let $z_{1,i}=\frac{y_{1,i}^{{}^{\prime }}}{max\{y_{1,i}^{{}^{\prime }}\}}$ and $z_{2,i}=\frac{y_{2,i}^{{}^{\prime }}}{max\{y_{2,i}^{{}^{\prime }}\}}$ make sets {*z*_1,*i*_} and {*z*_2,*i*_} invariant to different scaling of input models.

**Fig 3:**
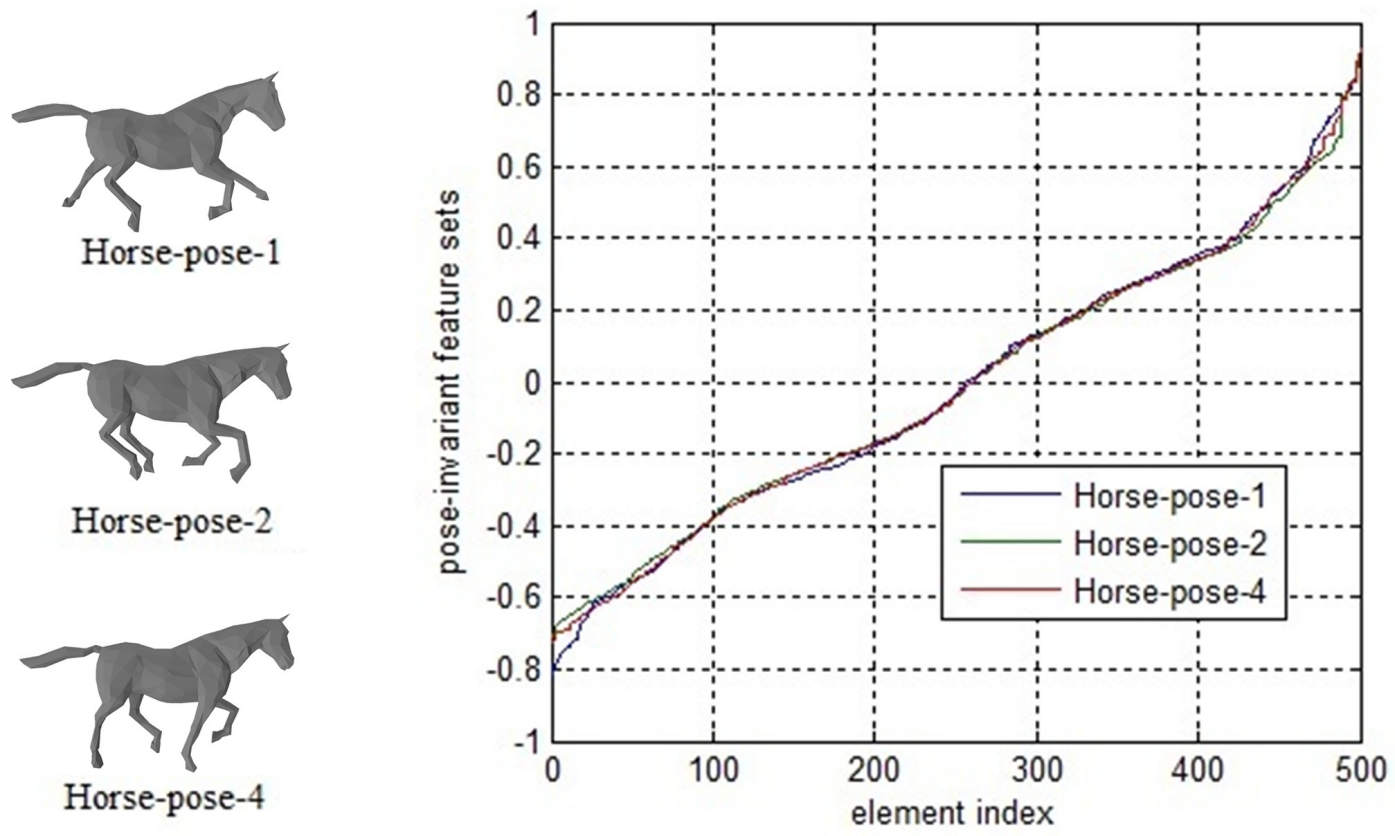
Our pose-invariant feature sets for 3 horse models with different poses. The feature set is defined in Section 3.4. The horizontal axis represents the *i*th element in each feature set. The vertical axis represents the corresponding *z*_*i*_ in each feature set.

We use the sets {*z*_1,*i*_} and {*z*_2,*i*_} as our pose-invariant feature sets of models *M*_1_ and *M*_2_ to measure the similarity between them. Benefiting from using geodesic distances, our feature sets are invariant to different poses of 3D models. Fig 3 shows the pose-invariant feature sets for three horse models with different poses. The three models all contain 500 vertices. From Fig 3, we can see that their feature sets are almost equal, although their poses are different.

### Measure similarities of mesh models using pose-invariant feature sets

For two different models *M*_1_ and *M*_2_, whose feature sets {*z*_1,*i*_} and {*z*_1,*i*_} (*i* = 1, …, *n*) are obtained respectively in previous steps, we define the similarity measure *d* as follows:

$$\begin{array}{c} d=min\{\frac{1}{n}\sum_{i=1}^{n}{\left(z_{1,i}-z_{2,i}\right)}^{2},\frac{1}{n}\sum_{i=1}^{n}{\left(z_{1,i}+z_{2,n+1-i}\right)}^{2}\}, \end{array}$$ (2)

where the purpose of the second item $\frac{1}{n}\sum_{i=1}^{n}{\left(z_{1,i}+z_{2,n+1-i}\right)}^{2}$ in the definition (2) is to make *d* invariant to reflection.

Before the model retrieval process start, all models to be retrieved are stored in the database and indexed by their feature sets off-line. When a query model is inputted by the user, the feature set of it is computed on-line and a searching process is carried out by comparing feature sets to find similar models of the query model in the database.

## Experimental results and analyzation

An ideal model matching algorithm for non-rigid models should be invariant to pose, scaling, and sampling as well as to rotation and translation. Meanwhile, that will be much better if it is robust to noise. In other words, the similarity measure *d* between any two models with different poses, scaling, or sampling density but representing the same object should be less than models representing different objects when measured with the algorithm.

**Fig 4:**
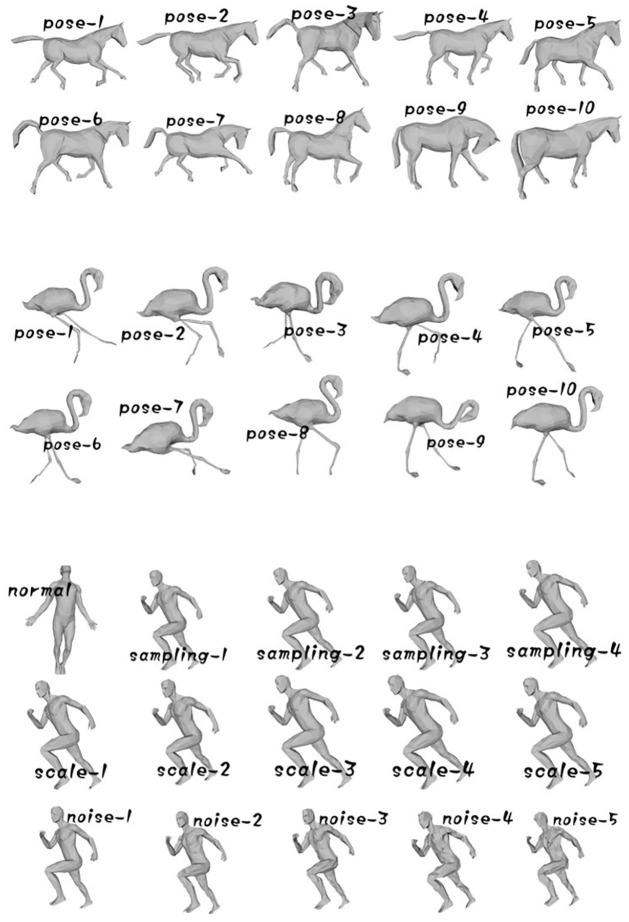
The model dataset used to test our algorithm. It contains 10 horse models with different poses, 10 flamingo models with different poses, and 15 human models with different sampling rates, scaling, and noise levels.

To verify that our algorithm is effective to measure the similarity between models, a model dataset (the testing dataset) is prepared to test the algorithm. The dataset contains 35 mesh models, as shown in Fig 4. All models in the dataset can be classified into 3 categories according to their contents. The first category is 10 models representing different poses of a horse. The second one is another 10 models representing different poses of a flamingo. The last one is a more complicated category which contains 15 human models with different sampling, scaling, or noise levels. Here, a bigger level number after “sampling”, “scaling”, or “noise” means the model has a higher sampling density, a bigger scaling rate, or more noises.

### Pose invariance testing

To test whether our algorithm is invariant against different poses, 5 models (horse-pose-1, horse-pose-3, flam-pose-2, flam-pose-4, and flam-pose-5) are selected from the testing dataset as query models, and the left 30 models are used as models to retrieve. The retrieval results obtained by using our algorithm are shown in Table 1.

**Table 1:** The query results of pose invariance testing.

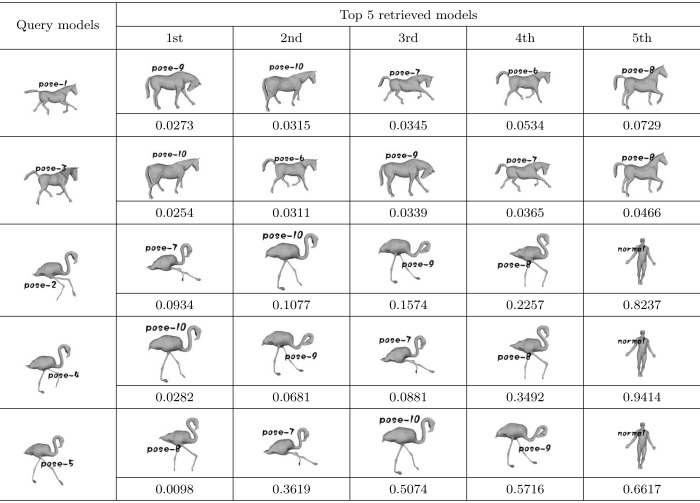

As described in Table 1, the models in the first column are query models selected from our model dataset. The top 5 most similar models (with least *d*) retrieved by our algorithm are listed in the remaining columns, and the corresponding similarity measures *d* are listed below the retrieved models. From Table 1, we can see that the models representing the same object with the query models are firstly retrieved with our algorithm, although their poses are different. By using geodesic distance instead of Euclidean distance, our similarity measures *d* between models representing different poses of the same objects are very small. And the similarity measures *d* between models representing different objects are much bigger. Therefore, we can demonstrate that our algorithm is pose-invariant through Table 1.

### Scaling invariance testing

To verify whether our algorithm is scaling-invariant, 3 models (human-scale-1, human-scale-3, human-scale-5) with different scaling are selected from the testing dataset as query dataset and another 22 models (including human-scale-2, human-scale-4, human-noise-2, human-noise-4, human-sampling-1, human-sampling-2, human-sampling-3, human-sampling-4, human-sampling-5, human-noise-1, human-noise-2, human-noise-3, human-noise-4, human-noise-5, 10 flamingo models and 10 horse models) are selected as models to retrieve. The retrieval results obtained by using our algorithm are shown in Table 2. From Table 2, we can see that our algorithm firstly retrieves human models with different scales. Moreover, the similarity measures *d* between models representing different scalings of the same objects are much smaller than other variants. Therefore, it means that our algorithm is scaling-invariant. Therefore, it means that our algorithm is scaling-invariant.

**Table 2:** The query results of scaling invariance testing.

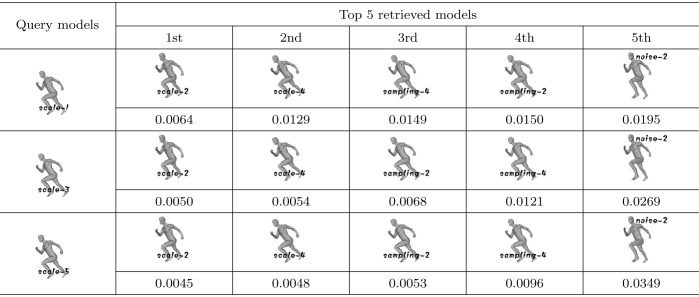

### Sampling invariance testing

To test whether our metric is sampling-invariant, 2 models (human-sampling-1, human-sampling-3) are used as query models. The remaining models in the testing dataset are used as models to be retrieved. Table 3 shows the query results of sampling invariance testing. From Table 3, we can see that although the human-sampling-2 model and human-sampling-4 model are not firstly retrieved, they still occur in the top 5 retrieved models. Note the other models in the top 5 retrieved models are all human models, which are scaled or noise-added rather than horse models or flamingo models. Therefore, we think that sampling only slightly affects the query results, and our algorithm is sampling-invariant.

**Table 3:** The query results of sampling invariance testing.

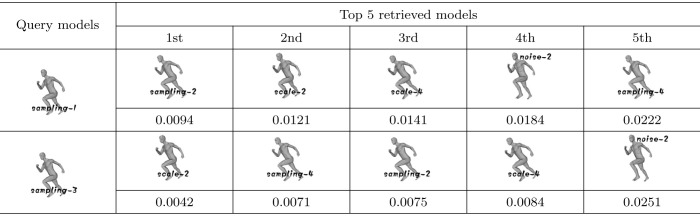

### Robustness testing

To test whether our algorithm is robust to noises added to models, 3 models (human-noise-1, human-noise-3, human-noise-5) are used as query models and the remaining models in the testing dataset are used as models to retrieve. Table 4 shows the query results of robustness testing. From the table, we can see that although the query models are added with some noises, the top 5 retrieved models are still human models. Therefore, our algorithm is robust to noise.

**Table 4:** The query results of robustness testing.

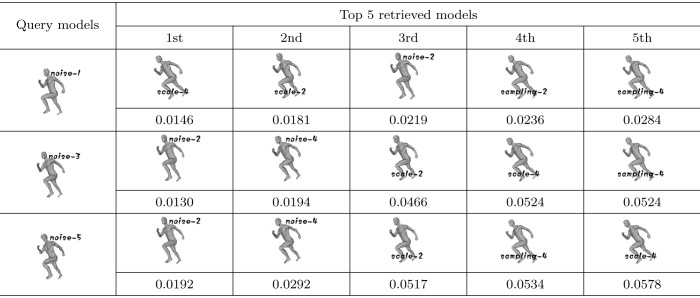

Through these experimental results, we can demonstrate that although 3D models representing the same object have different poses, scales, samples, or be added noises, our measures *d* between 3D models representing the same objects are much smaller than those between 3D models representing the different objects.

### Comparison with other 3D model retrieval methods

**Fig 5:**
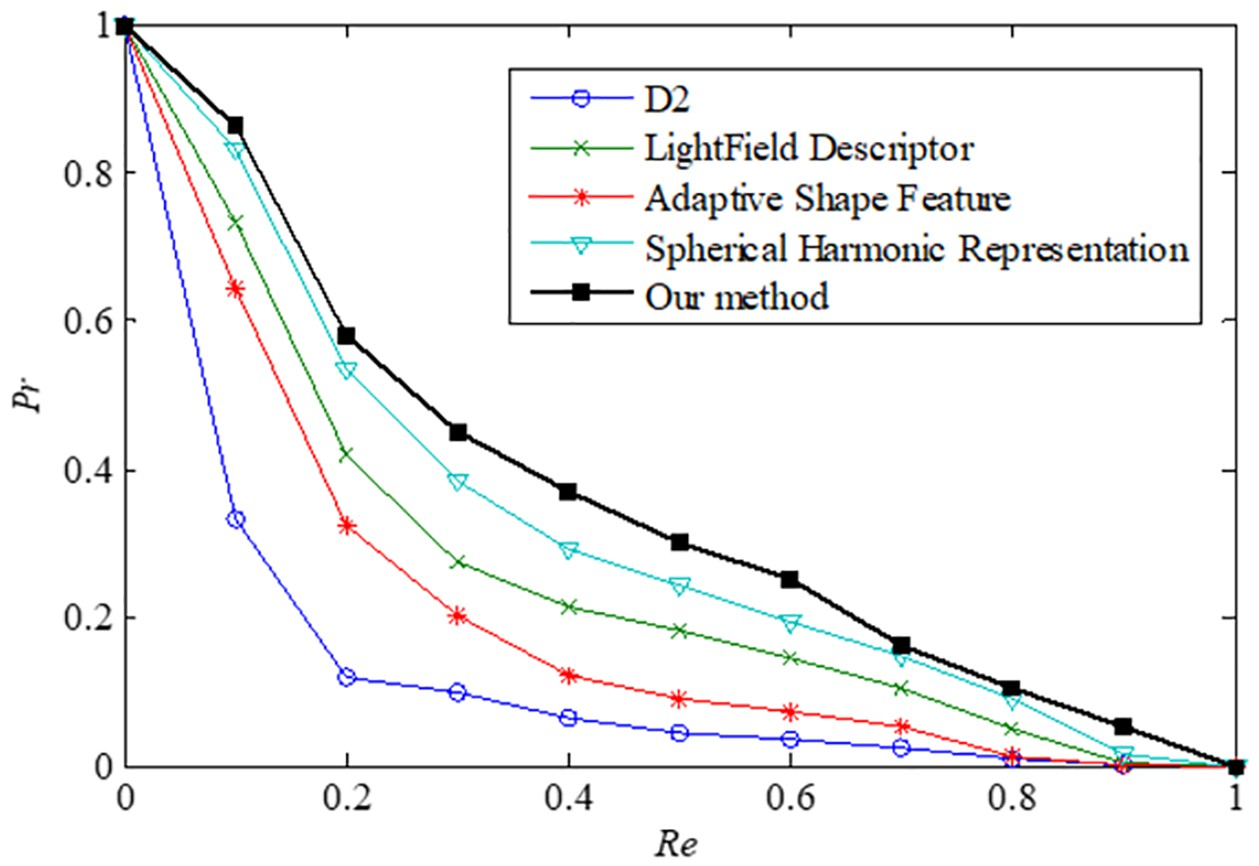
Precision vs. recall curves of our method and 4 other methods on our testing dataset. The horizontal axis represents recall rate, and the vertical axis represents precision rate. Higher curves represent better performance.

To compare the effectiveness of our method with other 3D model retrieval methods, experiments have been conducted on the testing dataset. Four other algorithms, including D2 [10], Adaptive Shape Feature [11], LightField Descriptor [12], Spherical Harmonic Representation [13], are implemented to compare the retrieval results. The performance is measured by recall and precision. The recall value *Re* and the precision value *Pr* are defined as follows,

Re=N/T,Pr=N/K,

where *N* is the number of relevant models retrieved, *T* is the total number of relevant models in the dataset, and *K* is the total number of retrieved models. The experimental results are presented in Fig 5. The area under the precision and recall curve (AUC) is a measurement showing how good the retrieval method is. The higher the AUC is, the greater the method can achieve. The results show that our method performs better than the other methods for our testing dataset.

**Fig 6:**
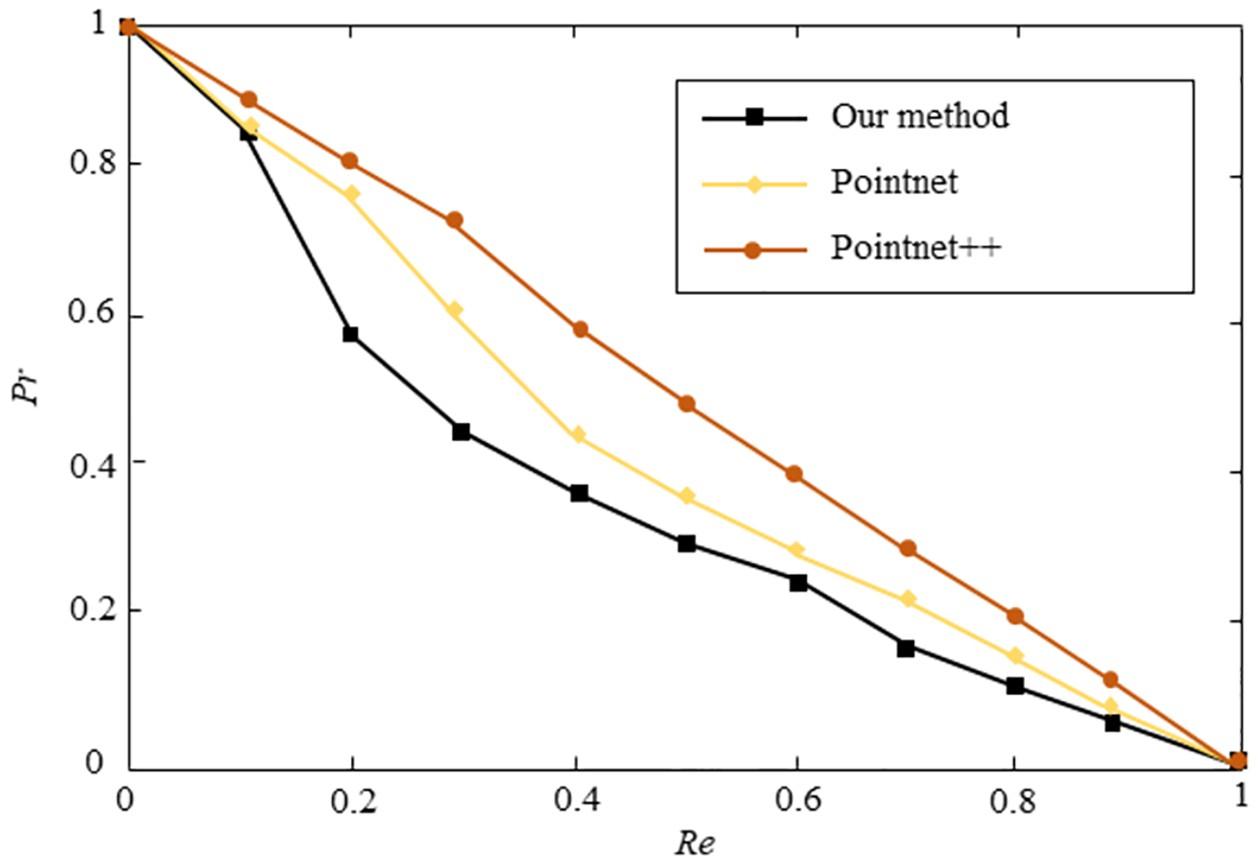
Performance of our method, PointNet, and PointNet++ when the training dataset contains 60 3D models. The horizontal axis represents recall rate, and the vertical axis represents precision rate. Higher curves represent better performance.

In addition, to compare the effectiveness of our method with the supervised learning algorithms, including PointNet and PointNet++, we evaluated the performance of PointNet and PointNet++ by using the same testing dataset. We used 60 3D models (20 flamingo models, 20 horse models, 20 man models) as the training dataset for PointNet and PointNet++ neural networks. Fig 6 shows the experimental results of PointNet, PointNet++, and our method. The results demonstrate that the testing results of PointNet and PointNet++ are slightly better than our method when the training dataset contains enough 3D models.

**Fig 7:**
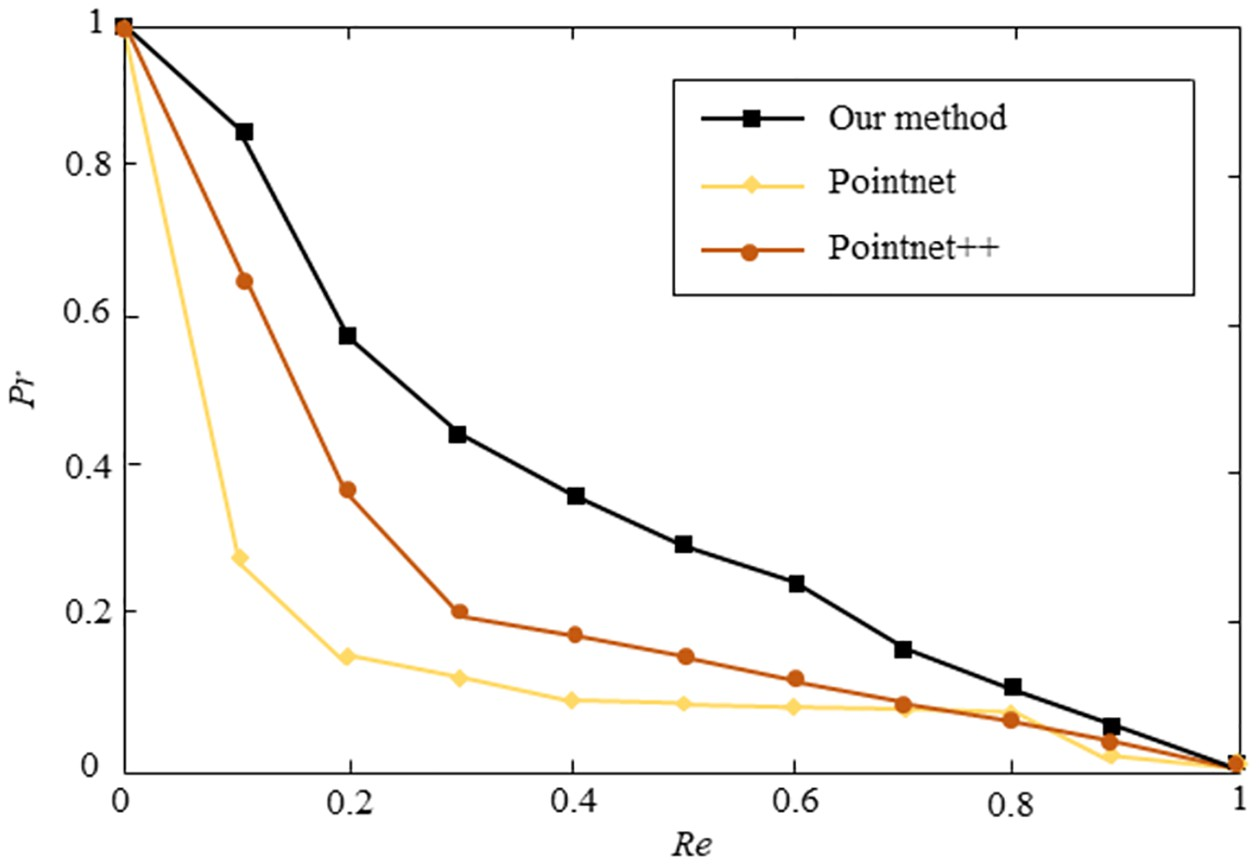
Performance of our method, PointNet, and PointNet++ when the training dataset contains 30 3D models. The horizontal axis represents recall rate, and the vertical axis represents precision rate. Higher curves represent better performance.

However, as shown in Fig 7, our method obtains better performance than PointNet and PointNet++ if only a small number of 3D models (10 flamingo models, 10 horse models, 10 man models) are provided in the training dataset. In other words, our method is more applicable in the absence of training data.

## Discussion and conclusions

In this paper, we present an effective algorithm for content-based 3D mesh model matching. By adopting Isomap, a pose-invariant feature set is constructed for each model. Then the similarities between models are measured by comparing their feature sets. Experimental results show that the algorithm is invariant to poses, scaling, rotation, and sampling. And also, it is robust to noise data. Additionally, tests show that our algorithm performs better than several referenced 3D model matching methods on our testing dataset. In future work, we plan to extend the algorithm to deal with 3D models contain multiple connected components.

## Supporting information

S1 Data (RAR)
